# IL‐22 produced by Th22 cells aggravates atherosclerosis development in ApoE^−/−^ mice by enhancing DC‐induced Th17 cell proliferation

**DOI:** 10.1111/jcmm.14967

**Published:** 2020-02-05

**Authors:** Lei Shi, Qingwei Ji, Ling Liu, Ying Shi, Zhengde Lu, Jing Ye, Tao Zeng, Yan Xue, Zicong Yang, Yu Liu, Jianyong Lu, Xinshun Huang, Qiuwen Qin, Tianzhu Li, Ying‐zhong Lin

**Affiliations:** ^1^ Department of Cardiology The People's Hospital of Guangxi Zhuang Autonomous Region Nanning China

**Keywords:** atherosclerosis, dendritic cells, IL‐22, inflammation, smooth muscle cells, Th22 cells

## Abstract

Th22 cells are a novel subset of CD4^+^ T cells that primarily mediate biological effects through IL‐22, with both Th22 cells and IL‐22 being closely associated with multiple autoimmune and chronic inflammatory diseases. In this study, we investigated whether and how Th22 cells affect atherosclerosis. ApoE^−/−^ mice and age‐matched C57BL/6J mice were fed a Western diet for 0, 4, 8 or 12 weeks. The results of dynamic analyses showed that Th22 cells, which secrete the majority of IL‐22 among the known CD4^+^ cells, play a major role in atherosclerosis. ApoE^−/−^ mice fed a Western diet for 12 weeks and administered recombinant mouse IL‐22 (rIL‐22) developed substantially larger plaques in both the aorta and aortic root and higher levels of CD3^+^ T cells, CD68^+^ macrophages, collagen, IL‐6, Th17 cells, dendritic cells (DCs) and pSTAT3 but lower smooth muscle cell (SMC) α‐actin expression than the control mice. Treatment with a neutralizing anti–IL‐22 monoclonal antibody (IL‐22 mAb) reversed the above effects. Bone marrow‐derived DCs exhibited increased differentiation into mature DCs following rIL‐22 and ox‐LDL stimulation. IL‐17 and pSTAT3 were up‐regulated after stimulation with IL‐22 and ox‐LDL in cells cocultured with CD4^+^ T cells and mature DC supernatant, but this up‐regulation was significantly inhibited by IL‐6mAb or the cell‐permeable STAT3 inhibitor S31‐201. Thus, Th22 cell‐derived IL‐22 aggravates atherosclerosis development through a mechanism that is associated with IL‐6/STAT3 activation, DC‐induced Th17 cell proliferation and IL‐22–stimulated SMC dedifferentiation into a synthetic phenotype.

## 1 INTRODUCTION

Atherosclerosis (AS) is a highly complex and multifactorial disease, with current data showing that atherosclerosis is the leading cause of death among all human diseases. A growing body of evidence suggests that atherosclerosis is a chronic inflammatory disease that is closely related to innate and acquired immunity.^1^, ^2^ During each stage of the disease, progressive atherosclerotic lesions display T‐lymphocyte infiltration. These T cells interact with local macrophages and vascular smooth muscle cells (SMCs) and affect the expression of inflammatory factors. Inflammatory factors such as MCP‐1, tumour necrosis factor‐α (TNF‐α) and interleukin (IL)‐6, which are expressed by macrophages and T lymphocytes, facilitate SMC migration from the tunica media to the intimal or subendothelial space, resulting in the formation of a fibrous cap.^3^, ^4^, ^5^

Studies have indicated that the lymphocytes present in a plaque are primarily CD4^+^ T cells,^6^, ^7^ which can be roughly divided into Th1, Th2, Th17 and regulatory T (Treg) cell lineages. The Th1 cell‐mediated immune response has been shown to promote the development of AS.^8^, ^9^ Th17 cells, which are a powerful inflammatory factor, may play important roles in accelerating AS in specific environments,^10^, ^11^, ^12^, ^13^ whereas Treg cells are atheroprotective.^14^, ^15^ The overall role of the Th2 immune response during AS remains unclear.^16^, ^17^, ^18^ Dendritic cells (DCs) are the most potent antigen‐presenting cells (APCs) and express costimulatory molecules, such as CD80 and CD86. DCs possess a strong capacity to activate and promote T‐cell differentiation. These cells were demonstrated to drive CD4^+^T‐cell differentiation along both the Th1 and Th17 pathways,^19^, ^20^ which partially occurs through STAT3 phosphorylation.^21^, ^22^

The Th22 subset is a novel CD4^+^T‐cell subset that was discovered in humans in 2009 and is distinct from the Th1, Th2 and Th17 subsets.^13^, ^23^, ^24^ In 2012, Basu et al demonstrated that Th22 cells (CD4^+^ IL‐22^+^) are also present in mice and are the most important source of IL‐22 in mice.^25^ Basu demonstrated that in the early stages of inflammation, IL‐22 is primarily secreted by lymphatic tissue‐induced cells. In the middle and late stages of inflammation, IL‐22 is pre‐dominantly released by Th22 cells. These results indicate that Th22 cells and IL‐22 likely play an important role in chronic inflammatory diseases. The biological effects of Th22 cells occur through IL‐22, which, though previously considered a Th17 cytokine, belongs to the IL‐10 family and has effects that are primarily mediated through IL‐22‐IL‐22R1 interaction.^26^, ^27^ IL‐22 binding to IL‐22R1 pre‐dominantly activates the STAT3 pathway.^28^

In recent years, the relationship between Th22 cells and clinical disease has attracted increased attention. IL‐22 protects against multiple infections of the lungs and intestine as well as liver injury, but it also has pathogenic roles in rheumatoid arthritis and psoriasis.^29^, ^30^, ^31^, ^32^, ^33^ However, few studies have investigated the roles of Th22 cells and IL‐22 in AS. Our previous study revealed significant increases in the peripheral Th22 cell numbers and IL‐22 levels of patients with acute coronary syndrome (ACS) compared to patients with stable angina pectoris (SAP) and control patients, suggesting that the circulating Th22‐type response may have a potential role in the onset of ACS symptoms.^23^ In this context, we focused on elucidating whether and how Th22 cells and IL‐22 act on AS.

## 2 MATERIALS AND METHODS

### 2.1 Animal models and interventions

Eight‐week‐old homozygous ApoE^−/−^ male mice (in a C57BL/6J background) and age‐matched C57BL/6J mice with bodyweights ranging from 20 to 25 g were obtained from The Jackson Laboratory housed under specific pathogen‐free conditions and fed a Western‐type diet (Clinton/Cybulsky Rodent Diet D12108 with 1.25% cholesterol; Research Diets). Animal studies were performed in accordance with the National Institutes of Health Animal Care and Use guidelines, and all breeding and experiment protocols were undertaken after review and approval by the Animal Ethics and Experimental Committee of the People's Hospital of Guangxi Zhuang Autonomous Region.

Half of the ApoE^−/−^ mice and all of the C57BL/6J mice were weighed, killed and analysed at weeks 0, 4, 8 and 12 (eight mice at each time‐point, Figure S1). Half of the ApoE^−/−^ mice were randomized into four groups (eight mice in each group) and intraperitoneally injected with 2 μg of recombinant IL‐22 (rIL‐22), 1× PBS, 20 µg of an anti–IL‐22 monoclonal antibody (IL‐22 mAb) or an isotype mAb (all from R&D Systems) thrice a week for 12 weeks (Figure S1).

### 2.2 Tissue preparation and atherosclerosis lesion evaluation

At 0, 4, 8 and 12 weeks, the mice were killed, and blood was collected retro‐orbitally to assess the IL‐22, IL‐6, MCP‐1 and TNF‐α concentrations by enzyme‐linked immunosorbent assays (ELISAs). The spleen, aorta and heart of each mouse were rapidly removed after perfusion with PBS. The spleens were mechanically dissociated to isolate lymphocytes for analysis of Th1, Th17 and Th22 cell frequencies by flow cytometry. The hearts and aortas were snap‐frozen at −80°C for further analysis. Atherosclerotic lesions were measured by lipid staining. Hearts with an attached aortic root were snap‐frozen in OCT compound, and the aortic sinus cross‐sections (10 μm per section) were excised, fixed in 4% formalin and stained with oil red O. For further analysis, five sections from each mouse were obtained from five different locations (each separated by 100 μm), and the lipid deposits in the aorta were fixed and stained as described above. Before euthanasia, all mice were anesthetized with 3% chloral hydrate at 10 μL/g bodyweight. All images were captured by a single observer blinded to the experimental protocol and analysed with computer image analysis software (Image‐Pro Plus 6.0).

### 2.3 Cell preparation

Mice were killed at 0, 4, 8 and 12 weeks, and lymphocytes from the spleens were isolated. Immediately after being removed from the mice, the fresh spleens were gently crushed with a clear glass pestle in cold PBS, and the splenocytes were passed through a 100 mesh stainless steel screen to prepare single‐cell suspensions. The cells were extracted with an equal volume of Ficoll‐Hypaque and washed twice in PBS to isolate the splenic lymphocytes.

### 2.4 Flow cytometry

#### 2.4.1 Analysis of IL‐22–related T cells

Cells were resuspended in PBS at a density of 10^5^‐10^6^ cells/mL and incubated with a fluorescein isothiocyanate (FITC)‐conjugated antimouse CD4 antibody at 4°C for 30 minutes for cell surface staining. After surface staining, the cells were washed twice and resuspended in RPMI‐1640 medium (Gibco) supplemented with 10% heat‐inactivated foetal calf serum (FCS; Gibco, BRL), also referred to as complete medium, and then transferred to 24‐well plates. The lymphocytes were stimulated with 2 μL of Cell Stimulation Cocktail (plus protein transport inhibitors, eBioscience). The cells were incubated at 37°C under a 5% CO_2_ atmosphere. After 4 hours of culturing, the contents of the wells were transferred to sterile 5‐mL tubes, where the cells were washed, fixed and permeabilized. Next, the cells were stained with allophycocyanin (APC)‐labelled anti‐interferon (IFN)‐γ mAbs, phycoerythrin‐Cy7 (PE/Cy7)‐labelled anti–IL‐17A mAbs and phycoerythrin (PE)‐labelled anti–IL‐22 mAbs according to the manufacturer's instructions (all of the mAbs were from eBioscience) to detect Th1, Th17 and Th22 cells. Isotype controls were used to enable correct compensation and to confirm the antibody specificity. The stained cells were analysed by flow cytometric analysis using a FACScan flow cytometer equipped with CellQuest software (BD Bioscience Pharmingen). Th1, Th17, Th22 and IL‐22–producing Th17 cells were defined as CD4^+^IFN‐γ^+^, CD4^+^IL‐17^+^, CD4^+^IFN‐γ^−^IL‐17^−^IL‐22^+^ and CD4^+^IFN‐γ^−^IL‐17^+^IL‐22^+^ T cells, respectively.

#### 2.4.2 Analyses of mature DCs

Cells were stained with antibodies against CD11c, CD86 and MHC‐II to study DC surface markers according to the manufacturer's protocols. The DC surface marker levels were measured by flow cytometry using the following antibodies: a FITC‐labelled anti‐CD11c antibody, a Brilliant Violet 421 (BV421)‐labelled anti‐CD86 antibody and a PE‐labelled anti–MHC‐II antibody (all of the antibodies were purchased from BioLegend Company).

### 2.5 ELISA

The serum concentrations of IL‐22, IL‐6, MCP‐1 and TNF‐α were measured by ELISAs according to the manufacturer's instructions (ELISA kits for IL‐6, MCP‐1 and TNF‐α, Neobioscience Technology; ELISA kits for IL‐22, BD Biosciences). The intra‐ and interassay coefficients of variation for all ELISAs were <5% and <10%, respectively. All assays were performed in triplicate.

### 2.6 Immunohistochemistry and Masson trichrome staining

Hearts with an attached aortic root were snap‐frozen in OCT compound and then cut into 4‐μm‐thickaortic sinus cross‐sections. Four cross‐sections were analysed from each mouse to quantitate the levels of IL‐22, IL‐22R1, macrophages, T cells, vascular SMCs and collagenin in the lesions. For immunohistochemical analyses, the sections were fixed with formalin and pre‐treated with formic acid, followed by endogenous peroxidase blocking with 2% normal goat serum and incubation with primary antibodies, including a rabbit antimouse IL‐22 antibody (1:500 dilution), a rabbit antimouse IL‐22R1 antibody (1:25 dilution), a rabbit antimouse CD3 antibody (1:500 dilution), a rabbit antimouse CD68 antibody (1:1000 dilution) and a rabbit antimouse SMC α‐actin antibody (1:100 dilution) (all of the Abs were purchased from Abcam) overnight at 4°C. The sections were washed the following day and then incubated for 1 hour with the appropriate goat anti‐rabbit HRP‐conjugated secondary antibodies (1:1000). Visualization of the antigen of interest was achieved after 3 minutes of exposure to diaminobenzidine, and the reaction was terminated with water. The results were observed under a 40× light microscope. The content of each lesion occupied by collagen was determined after Masson trichrome staining. All images were captured by a single observer blinded to the experimental protocol and analysed with computer image analysis software (Image‐Pro Plus 6.0).

### 2.7 Cell culture and intervention

Bone marrow‐derived DCs generation: The bone marrow was isolated from the femurs and tibiae of 8‐week‐old male ApoE^−/−^ mice (n = 8). The bone marrow cells were resuspended in RPMI‐1640 supplemented with 10% FCS (Gibco), 100 U/mL penicillin and streptomycin, and 20 ng/mL recombinant murine IL‐4 and GM‐CSF (BD Biosciences). On day 3, non‐adherent cells were carefully washed away, and the remaining cells were retreated with IL‐4 and GM‐CSF. On day 5, half of the culture medium was replaced with medium under the same conditions. Bone marrow‐derived DCs were collected and purified with CD11c microbeads and MACS (Miltenyi Biotec) on day 7 (purity > 95%) and divided into four groups according to the different interventions: (a) PBS; (b) rIL‐22 (100 ng/mL, R&D Systems); (c) ox‐LDL (50 μg/mL, Yiyuan Biotec); and (d) ox‐LDL^+^ rIL‐22 (100 ng/mL and 50 μg/mL, respectively). Every group had 5 wells with 1 × 10^6^ cells/well. After 24 hours of stimulation, the supernatants were stored at 4°C, and the DCs were collected for RT‐PCR analysis of the MHC‐II, CD68 and CD80 mRNA levels. After detection, the supernatant of the group with the highest level of mature DCs was retained for the subsequent analysis.

CD4^+^ T‐cell purification: Spleens (n = 8) from 8‐week‐old male ApoE^−/−^ mice were removed and used to prepare single‐cell suspensions. CD4^+^ cells were magnetically separated using Anti‐Mouse CD4 Magnetic Particles—DM (BD Biosciences) according to the manufacturer's instructions (purity > 95%). Purified CD4^+^ T cells were resuspended in the supernatant described above and divided into eight groups according to the different interventions: (a) PBS; (b) rIL‐22 (100 ng/mL); (c) rIL‐22+IL‐6mAb (100 and 10 ng/mL, respectively); (d) rIL‐22+S31‐201 (100 ng/mL and 10 μmol/L, respectively); (e) ox‐LDL (50 μg/mL); (f) ox‐LDL+rIL‐22 (50 μg/mL and 100 ng/mL, respectively); (g) ox‐LDL+rIL‐22+IL‐6mAb (50 μg/mL, 100 ng/mL and 10 ng/mL, respectively); and (h). ox‐LDL+rIL‐22+S31‐201 (50 μg/mL, 100 ng/mL and 10 μmol/L, respectively). Each group had 5 wells with 2 × 10^5^ cells/well. The cells were cultured at 37°C under an atmosphere with 5% CO_2_ for 72 h in RPMI‐1640 medium (Gibco) under Th17‐polarizing conditions (TGF‐β, 1 ng/mL; IL‐23, 50 ng/mL; anti‐IFN‐γ, 10 µg/mL; and anti‐IL‐4, 10 µg/mL; all from eBioscience, San Diego, CA) without IL‐6. After 3 days of culturing, the cells were collected for RT‐PCR and Western blot.

### 2.8 Real‐time quantitative reverse transcription‐polymerase chain reaction (RT‐PCR)

Total RNA was extracted from the aortas and cells with TRIzol reagent (Invitrogen) according to the manufacturer's instructions for analysis of the IL‐22, IL‐22R1, CD80, CD86 and IL‐17 mRNA levels. After reverse transcription was performed using a first‐strand cDNA synthesis kit (TaKaRa Biotechnology), the resultant cDNA was used as a template to perform real‐time PCR with the Applied Biosystems StepOne Real‐Time PCR System (Applied Biosystems) and a SYBR Green real‐time PCR master mix kit (TaKaRa Biotechnology). Each sample was analysed in triplicate, and gene expression was normalized to β‐actin expression. The gene expression levels were then calculated as the difference (ΔCt) between the Ct value of the target gene and that of β‐actin. The results are presented as the relative fold change compared with the 0‐week C57BL/6J mice (assigned a value of 1) using the standard 2^−ΔΔCt^ method. The primer sequences for the IL‐22, IL‐22R1, MHC‐II, CD80, CD86, IL‐17 and β‐actin genes are shown in Table 1.

**Table 1:** Primer sequences for RT‐PCR

Gene (mouse)	Forward (5′‐3′)	Reverse (5′‐3′)
rL‐22	CAACTTCCAGCAGCCATACA	GTTGAGCACCTGCTTCATCA
IL‐22R1	TGCTGGTTAGTTGTATGTC	ATGGAGGAGTGCTGCTTA
rIL‐17	GCTGACCCCTAAGAAACCCC	GAAGCAGTTTGGGACCCCTT
CD80	TCAGTTGATGCAGGATACACCA	AAAGACGAATCAGCAGCACAA
CD86	TCAATGGGACTGCATATCTGCC	GCCAAAATACTACCAGCTCACT
β‐actin	ACCCAC ACTGTGCCCATCTAC	AGC CAA GTC CAG ACG CAG G

### 2.9 Western blot analysis

Total protein was extracted from the aortas and cells and then detected with a BCA Protein Assay kit (Thermo Fisher Scientific). Protein samples (20 μg) were separated by electrophoresis on 8% Laemmli sodium dodecyl sulphate polyacrylamide gels. Equal amounts of protein per lane were electrophoretically transferred onto Immobilon‐FL PVDF membranes (Millipore). Then, the membranes were blocked with 5% non‐fat milk and incubated with anti‐STAT3 (1:1000; Cell Signaling Technology), anti‐phosphorylated STAT3 (1:2000; Cell Signaling Technology) and anti‐GAPDH antibodies at 4°C overnight followed by incubation with a goat antimouse IgG HRP‐conjugated secondary antibody (1:5000, Abcam) at room temperature for 1 hour. The blots were visualized using a 2‐colour infrared imaging system (Odyssey; LI‐COR Biosciences).

### 2.10 Statistical analysis

All data are presented as the means ± SD. Multiple‐group comparisons were performed by ANOVA. Differences between two groups were compared by Student's *t* test, and *P* < .05 was considered significant.

## 3 RESULTS

### 3.1 Basic physiological parameters

**Figure 1:**
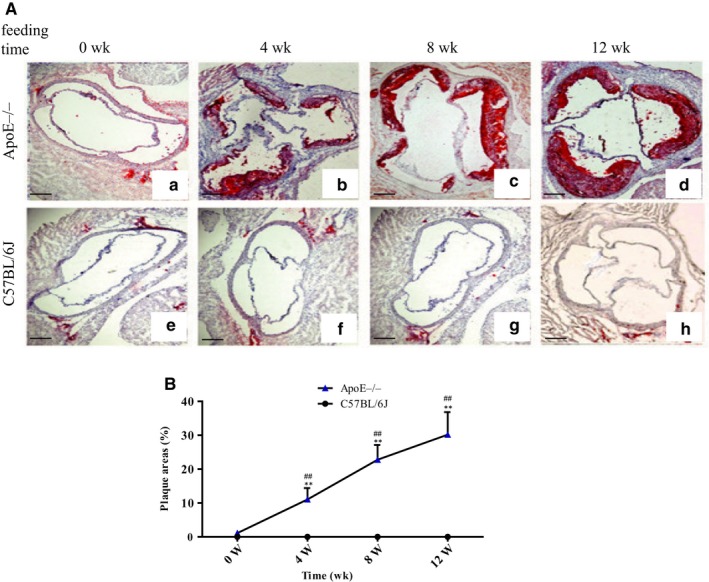
Atherosclerotic lesion area. A, Plaque areas in oil red O‐stained cryostat sections of the aortic roots from the ApoE^−/−^ mice and age‐matched C57BL/6J mice; scale bar = 200 μm (magnification, 10×). a‐d, ApoE^−/−^mice; e‐h, C57BL/6J mice. B, Atherosclerotic lesion area/lumina in the aortic root reported as percentages (n = 8). **P < .01 vs the age‐matched C57BL/6J mice. ^#^ *P* < .05 and ^##^ *P* < .01 vs different time‐points in the ApoE^−/−^mice. ▲, ApoE^−/−^ mice; ●, C57BL/6J mice

The bodyweight gain of the ApoE^−/−^ mice and age‐matched C57BL/6J mice fed a Western diet over 0, 4, 8 or 12 weeks showed no significant differences (ApoE^−/−^ mice vs C57BL/6J mice, n = 10; 0 weeks: 25.04 ± 1.25 g vs 24.42 ± 1.39 g; 4 weeks: 27.38 ± 1.17 g vs 27.48 ± 1.43 g; 8 weeks: 31.43 ± 2.06 g vs 31.21 ± 1.60 g; and 12 weeks: 34.49 ± 1.72 g vs 34.06 ± 1.65 g). The ApoE^−/−^ mice fed a Western diet showed prominent and continual progression of atherosclerotic lesions, and the atherosclerotic lesion size increased significantly as the feeding time increased (Figure 1 A and B). However, the C57BL/6J mice had no visible atherosclerotic lesions under the same conditions (Figure 1A).

### 3.2 Th22 cells are the major source of IL‐22 during atherosclerosis

**Figure 2:**
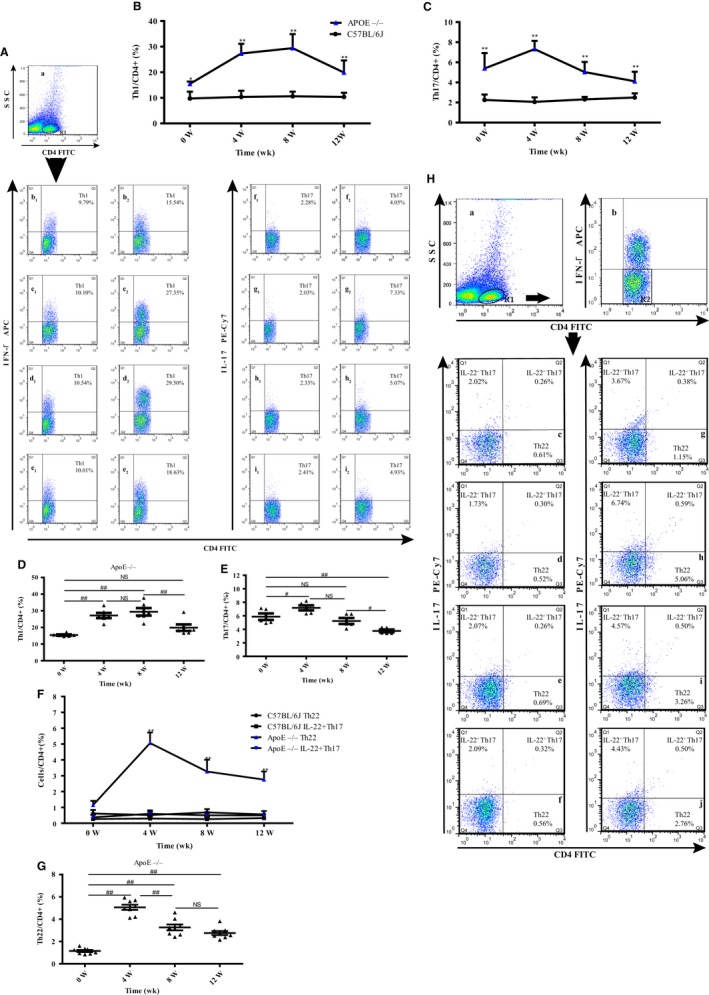
Proatherogenic and IL‐22–related CD4^+^ T cell frequencies in the spleen. A, (a) CD4^+^ T‐cell gating for flow cytometric analysis. (b1‐e1) The frequencies of Th1 cells in the C57BL/6J mice. b1: 0 wk; c1: 4 wk; d1: 8 wk; and e1: 12 wk. (b2‐e2) The frequencies of Th1 cells in the age‐matched ApoE^−/−^ mice. b2: 0 wk; c2: 4 wk; d2: 8 wk; and e2: 12 wk. (f1‐i1) The frequencies of Th17 cells in the C57BL/6J mice. f1: 0 wk; g1: 4 wk; h1: 8 wk; and i1: 12 wk. (f2‐i2) The frequencies of Th17 cells in the age‐matched ApoE^−/−^ mice. f2: 0 wk; g2: 4 wk; h2: 8 wk; and i2: 12 wk. The frequencies of (B) Th1 (CD4^+^ IFN‐γ^+^ T cells), (C) Th17 (CD4^+^ IL‐17^+^ T cells), (F) Th22 (CD4^+^ IFN‐γ‐IL‐17− IL‐22^+^ T cells) and IL‐22–producing Th17 (CD4^+^ IFN‐γ‐IL‐17^+^ IL‐22^+^ T cells) cells in the ApoE^−/−^ mice (n = 8) compared with the age‐matched C57BL/6J mice (n = 8) at all time‐points. The frequencies of (D) Th1, (E) Th17 and (G) Th22 cells in the ApoE^−/−^ mice compared at the different time‐points. (H) (a) CD4^+^ T‐cell gating for flow cytometric analysis. (b) Exclusion of IFN‐γ^+^ cells. (c‐f) The frequencies of Th22 cells in the C57BL/6J mice. c: 0 wk; d: 4 wk; e: 8 wk; and f: 12 wk. (g‐j) The frequencies of Th22 cells in the age‐matched ApoE^−/−^ mice. g: 0 wk; h: 4 wk; i: 8 wk; and j: 12 wk. *P < .05 and **P < .01 vs the age‐matched C57BL/6J mice. ^#^ *P* < .05 and ^##^ *P* < .01 vs different time‐points in the ApoE^−/−^mice. NS: no significance. ▲, ApoE^−/−^ mice; ●, C57BL/6J mice

To elucidate the role of Th22 cells in the development of AS, we detected proatherogenic and IL‐22–related CD4^+^ T cells with flow cytometry at 0, 4, 8 and 12 weeks. We observed that the proportions of Th1 (CD4^+^IFN‐γ^+^/CD4^+^ T cells), Th17 (CD4^+^IL‐17^+^/CD4^+^ T cells) and Th22 (CD4^+^IFN‐γ^−^IL‐17^−^IL‐22^+^/CD4^+^ T cells) cells were higher in the ApoE^−/−^ mice than the age‐matched C57BL/6J control mice at each time‐point (Figure 2A,H). The Th1 cell frequencies were dramatically higher in the ApoE^−/−^ mice than in the C57BL/6J control mice, especially at weeks 4 and 8 (Figure 2B). The Th17 and Th22 cell frequencies peaked at 4 weeks and then decreased but maintained significantly higher frequencies in the ApoE^−/−^ mice than in the C57BL/6J control mice at 12 weeks (Figure 2C,F). To further evaluate the trends of these changes, we compared the frequencies at 4, 8 and 12 weeks with those at 0 week. The Th1 cell frequency remained relatively high at 4 and 8 weeks but decreased to its initial level at 12 weeks (Figure 2D). The Th17 cell frequency nearly dropped to its initial level at 8 weeks and continued to decline at 12 weeks (Figure 2E). The Th22 cell frequency peaked at 4 weeks and remained relatively high at 12 weeks (Figure 2G). These results may indicate that Th1 cells play a crucial role in the middle stage of AS. Th17 cells exert a stronger influence on AS at an early stage, while Th22 cells may play an important role during the middle‐late stage of AS. To elucidate the role of Th22 cells, we further assessed the dynamic changes in the proportion of IL‐22–producing Th17 cells (CD4^+^IFN‐γ^−^IL‐17^+^IL‐22^+^/CD4^+^ T cells) (Figure 2H). The levels of IL‐22–producing Th17 cells were higher in the ApoE^−/−^ mice than in the control mice at all time‐points, but no significant differences were observed between these groups of mice (Figure 2F). In addition, no significant differences were observed among the frequencies at 4, 8, 12 and 0 weeks in the ApoE^−/−^ mice (Figure 2F). These results further demonstrated that among the known CD4^+^ cells, Th22 cells, which secrete most of the IL‐22, play a major role in AS.

### 3.3 IL‐22 and IL‐22R1 are expressed in mouse atherosclerotic plaques, and their expression levels are increased in ApoE^−/−^ mice

**Figure 3:**
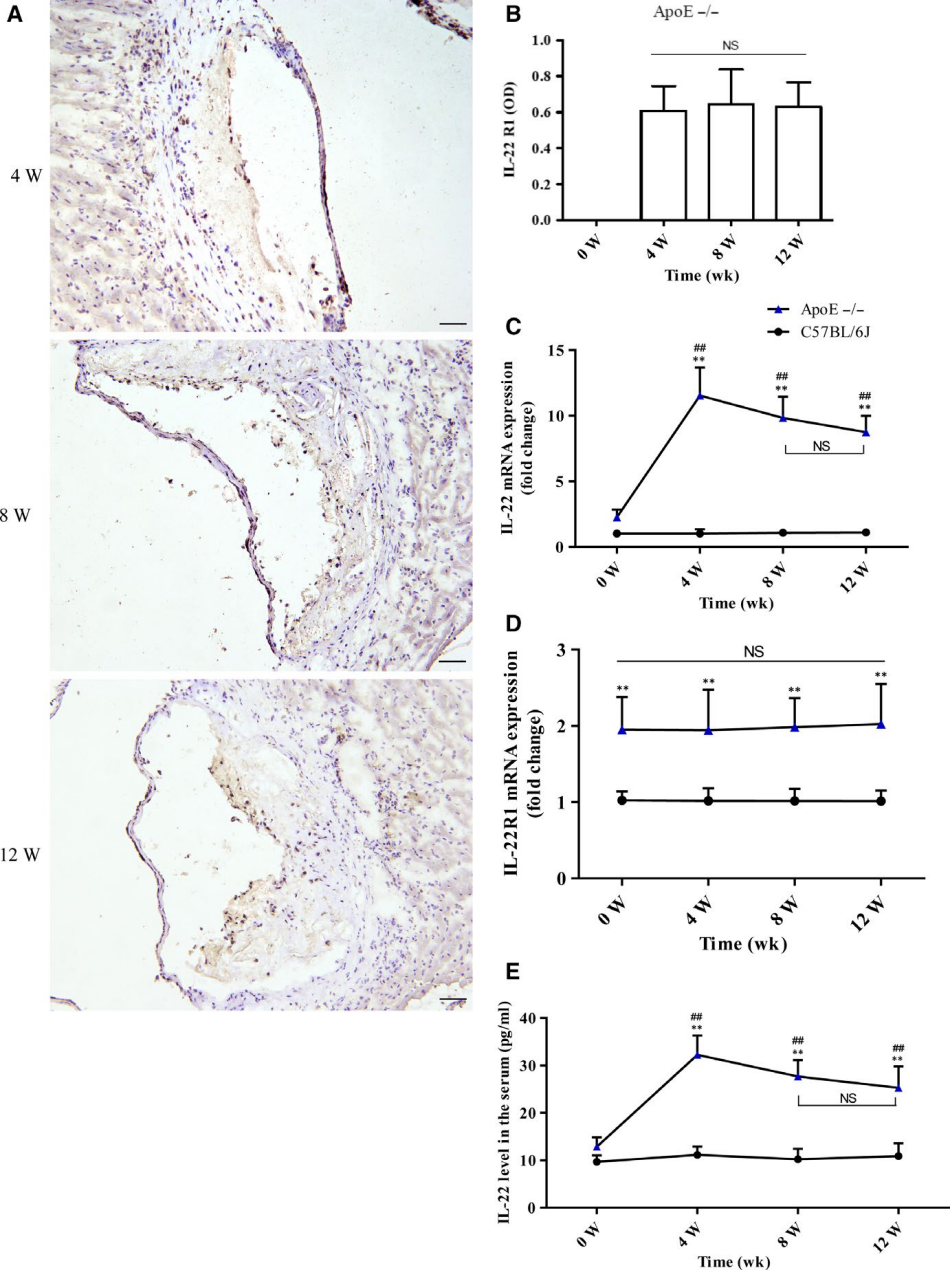
IL‐22 and IL‐22R1 are expressed in aortic tissue. A, The expression of IL‐22 receptor 1 in atherosclerotic plaques is shown. Black arrows show IL‐22R1–expressing cells; scale bar = 100 μm (magnification, 20× and 40×). B, The expression of IL‐22 R1 showed no significant differences between the groups (n = 8) with varying degrees of atherosclerotic lesions. (C and D) The mRNA levels of IL‐22 and IL‐22R1 in the aortas of the ApoE^−/−^ mice (n = 8) and the age‐matched C57BL/6J mice at different time‐points (n = 8). E, The serum concentrations of IL‐22 in the ApoE^−/−^ mice (n = 8) and the age‐matched C57BL/6J mice (n = 8) at different time‐points. **P < .01 vs the age‐matched C57BL/6J mice. ^##^ *P* < .01 vs different time‐points in the ApoE^−/−^mice. NS, no significance; ▲, ApoE^−/−^ mice; ●, C57BL/6J mice

As noted previously, IL‐22R is expressed in various non‐immune tissues, such as the skin, lungs and kidneys, and in cardiomyocytes. Through immunohistochemical analyses, we showed that IL‐22R1 is also expressed in mouse atherosclerotic plaques, and the expression levels of this molecule were similar among groups with varying degrees of atherosclerotic lesions (Figure 3A,B). To further explore the dynamic changes in IL‐22 and IL‐22R1 levels in AS, we dynamically assessed the aorta by real‐time PCR analyses. We observed that the mRNA levels of IL‐22 and IL‐22R1 were significantly higher in the ApoE^−/−^ mice than in the age‐matched C57BL/6J mice at all time‐points (Figure 3C,D). IL‐22R1 mRNA expression showed no significant differences at 0, 4, 8 and 12 weeks (Figure 3D). IL‐22 mRNA expression peaked at 4 weeks and remained relatively high at 12 weeks (Figure 3C), and ELISAs of the serum IL‐22 concentration exhibited a similar trend (Figure 3E).

### 3.4 IL‐22 aggravates atherosclerosis development

**Figure 4:**
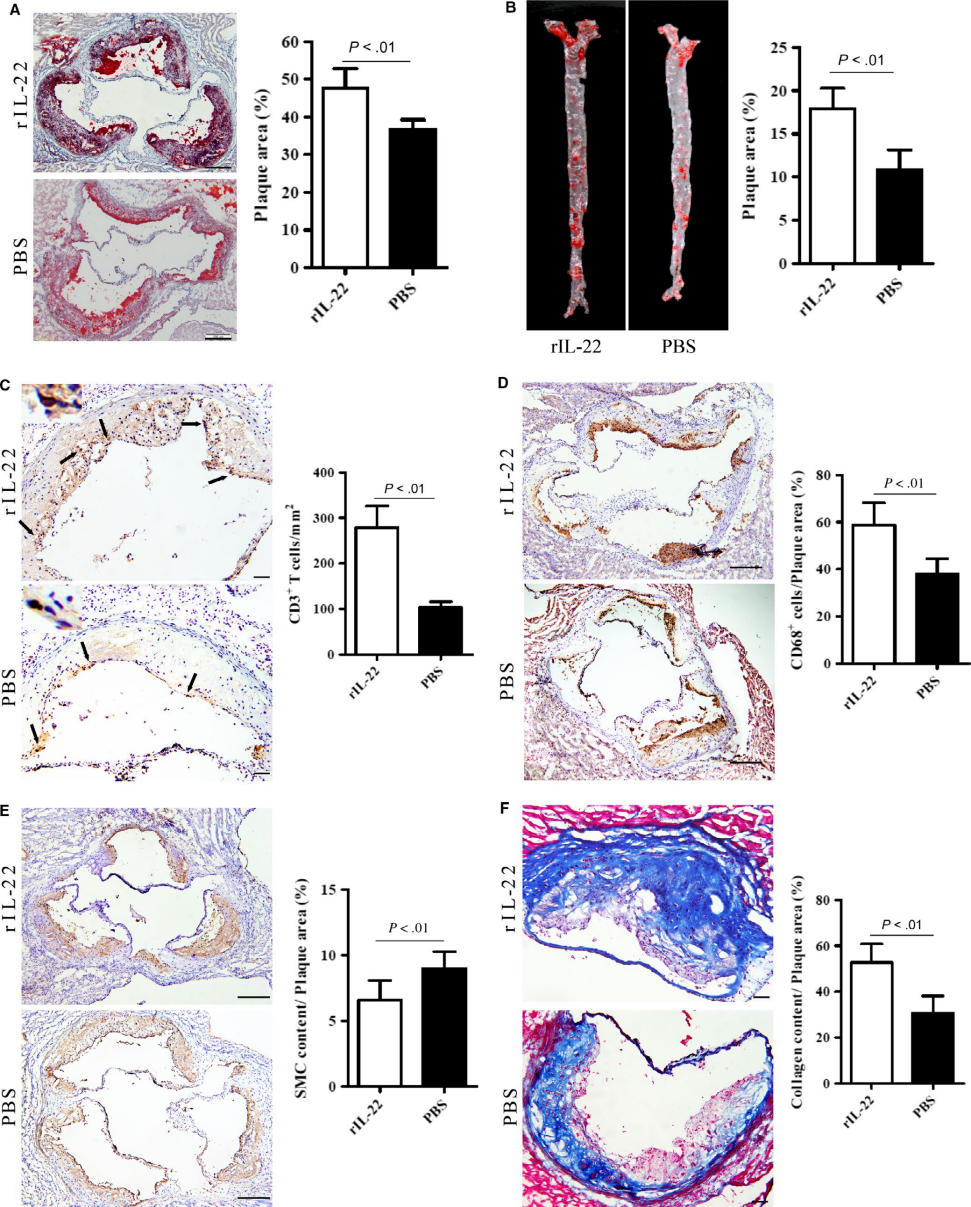
Mice treated with recombinant IL‐22 for 12 wk exhibit increased atherosclerotic plaque size, T cell and macrophage accumulation and collagen content but decreased SMC α‐actin content. A, The plaque area in cryostat sections stained with oil red O and the atherosclerotic lesion area/lumina in the aortic root reported as percentages (n = 8); scale bar = 200 μm (magnification, 10×). B, The plaque area in the aorta stained with oil red O and the quantification of aortic lesion area/total aortic area (n = 8). C, Sections stained with specific antibodies to detect CD3^+^ T cells and the quantitative analysis of the numbers of CD3^+^ T cells in atherosclerotic plaques (n = 8); black arrows show CD3^+^ T cells; scale bar = 200 μm (magnification, 20×). (D) Sections stained with specific antibodies to detect macrophages (CD68^+^ cells) and the quantification of CD68^+^ area/plaque area (n = 8); scale bar = 200 μm (magnification, 10×). E, Sections stained with specific antibodies to detect smooth muscle cells and the quantification of SMC α‐actin + area/plaque area (n = 8); scale bar = 200 μm (magnification, 10×). F, Sections stained with Masson trichrome to detect collagen and the quantification of collagen content/plaque area (n = 8); scale bar = 200 μm (magnification, 20×)

To further evaluate the role of IL‐22 in the development of AS, we treated ApoE^−/−^ mice with rIL‐22 for 12 weeks. First, the plaque sizes in the aortic root and the whole aorta were analysed. We observed that the plaque area was significantly increased in the mice treated with rIL‐22 compared with those treated with PBS (Figure 4A). Additionally, the aortic plaque burden was substantially greater in the mice treated with rIL‐22 than in those treated with PBS (Figure 4B). These results indicated that IL‐22 is involved in plaque formation. This outcome was consistent with the results for CD3^+^ T cells and CD68^+^ macrophage infiltration into the plaques. The infiltration of both cell types was strongly increased in the rIL‐22–treated mice compared with the PBS‐treated controls (Figure 4C,D). In contrast, SMC α‐actin expression was reduced in the rIL‐22–treated group (Figure 4E). These results suggest that IL‐22 can exacerbate AS.

**Figure 5:**
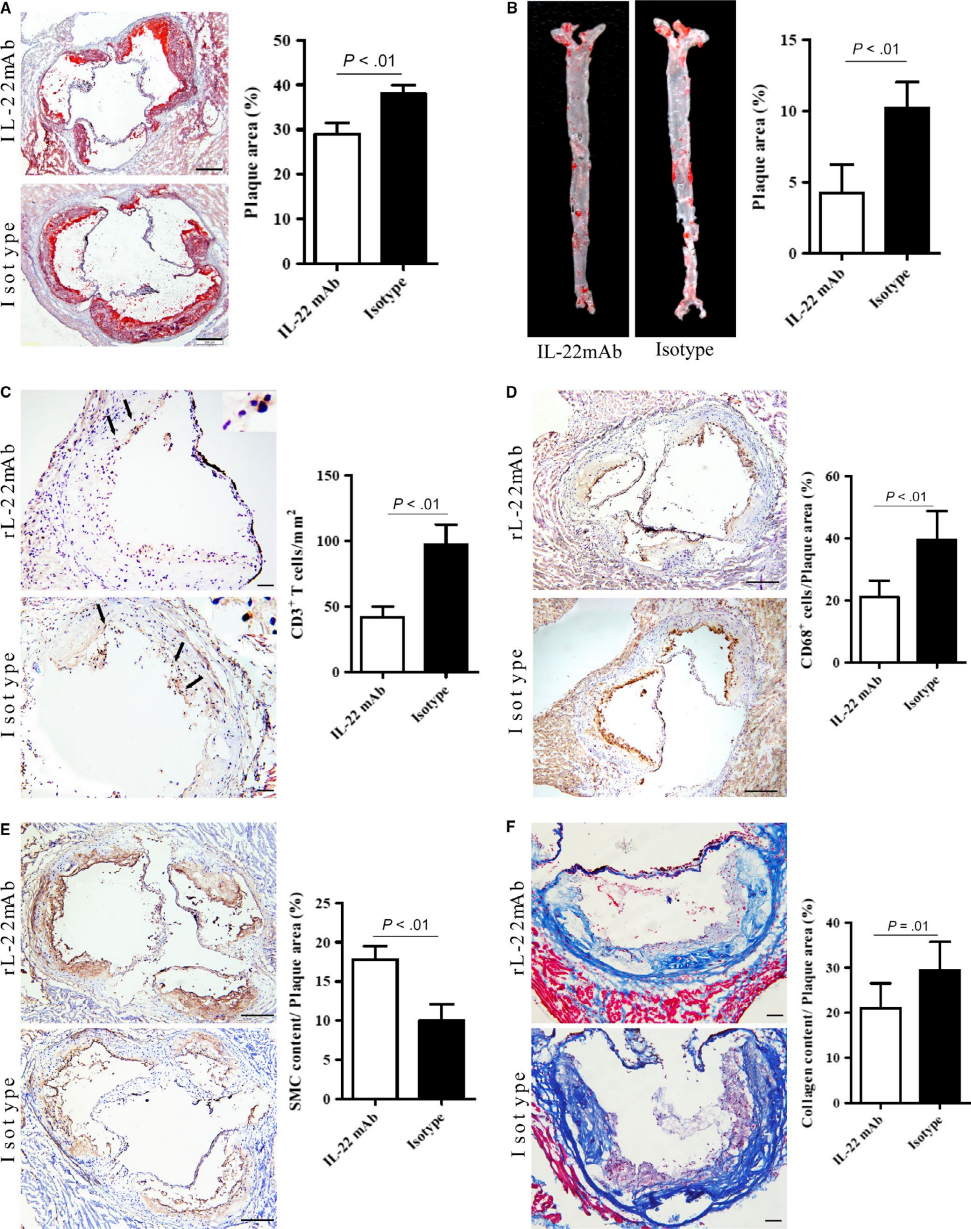
Neutralization of IL‐22 decreases atherosclerotic plaque size, T cell and macrophage accumulation and collagen content but increases SMC α‐actin content. A, The plaque area in cryostat sections stained with oil red O and atherosclerotic lesion area/lumina in the aortic root reported as percentages (n = 8); scale bar = 200 μm (magnification, 10×). B, The plaque area of the aorta stained with oil red O and the quantification of aortic lesion area/total aortic area (n = 8). C, Sections stained with specific antibodies to detect CD3^+^ T cells and the quantitative analysis of the numbers of CD3^+^ T cells in atherosclerotic plaques (n = 8); black arrows show CD3^+^ T cells; scale bar = 200 μm (magnification, 20×). D, Sections stained with specific antibodies to detect macrophages (CD68^+^ cells) and the quantification of CD68^+^ area/plaque area (n = 8); scale bar = 200 μm (magnification, 10×). E, Sections stained with specific antibodies to detect smooth muscle cells and the quantification of SMC α‐actin + area/plaque area (n = 8); scale bar = 200 μm (magnification, 10×). F, Sections stained with Masson trichrome to detect collagen and the quantification of collagen content/plaque area (n = 8); scale bar = 200 μm (magnification, 20×)

To further test the abovementioned conclusion, we used a neutralizer of IL‐22 (IL‐22 mAb) to block circulating IL‐22 in ApoE^−/−^ mice and observed that the plaque area in the aortic root was substantially reduced in the mice treated with IL‐22 mAb compared with those treated with an isotype control (Figure 5A). In addition, the aortic plaque burden was notably lower in the IL‐22 mAb‐treated ApoE^−/−^ mice than in the isotype mAb‐treated ApoE^−/−^ mice (Figure 5B). The areas occupied by CD3^+^ T cells and CD68^+^ macrophages in the plaques were also significantly decreased in the IL‐22 mAb‐treated mice compared with the isotype control‐treated mice (Figure 5C,D), and the SMC numbers were substantially increased (Figure 5E). These findings indicated that the neutralization of IL‐22 decreases the development of AS and that IL‐22 exacerbates this process.

However, neither the rIL‐22 nor the anti–IL‐22 mAb affected the bodyweight of ApoE^−/−^ mice fed a high‐fat diet (rIL‐22–treated group vs PBS‐treated group: n = 16; 33.59 ± 3.43 g vs 35.06 ± 2.63 g; *P* = .19; IL‐22 mAb‐treated group vs isotype control‐treated group: n = 16; 35.82 ± 3.71 g vs 34.22 ± 2.64 g; *P* = .17).

### 3.5 IL‐22 promotes the phenotypic dedifferentiation of SMCs

Given the observed trend of the variation in SMC α‐actin, we subsequently performed Masson trichrome staining to assess the collagen contents of plaques within the aortic roots. Surprisingly, the collagen content was significantly increased in the rIL‐22–treated group (Figure 4F) and decreased in the IL‐22 mAb‐treated group (Figure 5F) compared with their respective control groups. This finding was consistent with earlier results^34^ and indicated that IL‐22 contributes to the pathogenesis of AS by stimulating the dedifferentiation of SMCs from a contractile phenotype into a synthetic phenotype.

### 3.6 IL‐22 increases DC and Th17 cell numbers by activating IL‐6/STAT3

**Figure 6:**
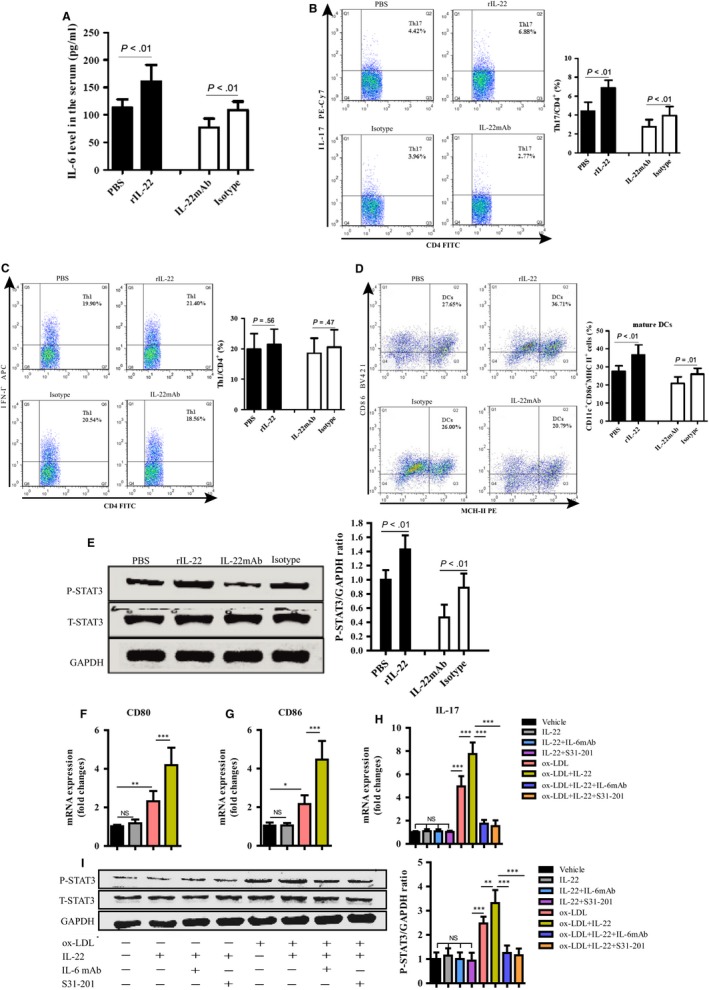
IL‐22 induces DC maturation and Th17 cell proliferation through the IL‐6/STAT3 pathway. A, ELISA showing an elevation in the serum IL‐6 concentration of ApoE^−/−^ mice treated with rIL‐22 (n = 8) and a reduction in the serum IL‐6 concentration of ApoE^−/−^ mice treated with IL‐22 mAb (n = 8) compared to their corresponding controls (n = 8). Flow cytometry showing elevations in the numbers of (B) Th17 cells, (C) Th1 cells and (D) DCs (CD11c+CD86+MHC‐II+) in rIL‐22–treated ApoE^−/−^ mice (n = 8) and decreases in IL‐22 mAb‐treated ApoE^−/−^ mice (n = 8) compared with their corresponding controls (n = 8). E, Western blot analysis showing a significant increase in the phosphorylation of STAT3 in the aorta of ApoE^−/−^ mice treated with rIL‐22 (n = 8) and a reduction in the aorta of ApoE^−/−^ mice treated with IL‐22 mAb (n = 8) compared to their corresponding controls (n = 8). F, The mRNA levels of CD80 in bone marrow‐derived DCs. G, The mRNA levels of CD80 in bone marrow‐derived DCs. H, The mRNA levels of IL‐17 in CD4^+^ T cells. I, Western blot for STAT3 phosphorylation levels in CD4^+^ T cells.*P < .05, **P < .01, ***P < .001, NS, no significance

To investigate the inflammatory mechanisms by which circulating IL‐22 may promote aggravated AS, we further evaluated proatherogenic cells in mouse spleens via flow cytometry and the concentration of proatherogenic inflammatory factors in the serum via ELISAs. We observed significant increases in the levels of IL‐6 and Th17 cells in the rIL‐22–treated ApoE^−/−^ mice and an strong decrease in IL‐22 mAb‐treated ApoE^−/−^ mice compared with the corresponding control mice (Figure 6A,B). However, these interventions had no impact on other proinflammatory factors, such as TNF‐α, IL‐1β and MCP‐1 (Table 2). We observed increased Th1 cell numbers in the rIL‐22–treated ApoE^−/−^ mice and decreased numbers in the IL‐22 mAb‐treated ApoE^−/−^ mice compared with the corresponding control mice. Nonetheless, no significant differences were observed among the groups (Figure 6C).

**Table 2:** Proatherogenic inflammatory factors in the serum

Factors (n = 8)	rIL‐22 vs PBS (pg/mL)	*P*	IL‐22 mAb vs Isotype (pg/mL)	*P*
IL‐6	160.10 ± 31.0 vs 112.86 ± 15.39	.00	77.05 ± 16.42 vs 110.15 ± 17.56	.00
TNF‐α	81.97 ± 14.44 vs 76.22 ± 13.68	.43	75.92 ± 12.81 vs 79.24 ± 14.6	.63
IL‐1β	103.95 ± 10.8 vs 100.43 ± 13.3	.57	97.10 ± 17.1 vs 99.09 ± 17.2	.82
MCP‐1	3,707.25 ± 779.63 vs 3,589.75 ± 835.62	.85	3,226.53 ± 894.65 vs 3,318.53 ± 969.63	.76

Th17 cells are regulated by cytokines secreted from APCs, especially DCs. In addition, the differentiation of Th17 cells is mediated by cytokines secreted by APCs, such as IL‐6.^35^ Therefore, we hypothesized that the enhanced IL‐6 and DC levels induced by pre‐treatment with IL‐22 may be responsible for the increased Th17 cell differentiation. Thus, we evaluated the numbers of DCs in the spleens of mice via flow cytometry. The flow cytometric analysis revealed a significant increase in the proportion of CD11c^+^CD86^+^MHC‐II^+^cells in the spleens of the rIL‐22–treated ApoE^−/−^ mice and a significant decrease in the spleens of the IL‐22 mAb‐treated ApoE^−/−^ mice (Figure 6D). Further Western blot analyses revealed that STAT3 phosphorylation was significantly increased in the IL‐22–treated mice compared with the PBS‐treated mice and decreased in the mice treated with IL‐22 mAb compared with those treated with an isotype control (Figure 6E). These results indicate that the mechanism by which IL‐22 promotes AS is associated with activated IL‐6/STAT3 and DC‐mediated induction of Th17 cell proliferation.

### 3.7 IL‐22 induces DC maturation and Th17 cell proliferation through the IL‐6/STAT3 pathway in vitro

Because we observed increased numbers of mature DCs and Th17 cells, as well as increased levels of IL‐6 and pSTAT3 in rIL‐22–treated ApoE^−/−^ mice fed a western diet, we conducted cell‐based experiments to further elucidate the underlying mechanisms, especially the downstream signalling pathways involved. First, we isolated bone marrow‐derived DCs from ApoE^−/−^ mice and pre‐treated/untreated them with rIL‐22 (100 ng/mL), followed by ox‐LDL stimulation (Figure S1). We observed that IL‐22 up‐regulated the expression of CD80 (Figure 6F) and CD86 (Figure 6G) in DCs after stimulation with ox‐LDL. Thus, IL‐22 can induce the maturation of BMDCs under high‐fat conditions. Subsequently, we retained the supernatant from Group d, which was treated with rIL‐22 and ox‐LDL, and used it to treat purified CD4^+^ T cells (Figure S1). Through pre‐treatment with rIL‐22 (100 ng/mL), IL‐6mAb (10 ng/mL) or/and S31‐201 (10 μmol/L), we elucidated the roles of IL‐22, IL‐6 and STAT3 in promoting Th17 cell proliferation. IL‐17 and STAT3 phosphorylation were up‐regulated after stimulation with IL‐22 and ox‐LDL, but this up‐regulation was significantly inhibited after treatment with IL‐6 mAb or the cell‐permeable STAT3 inhibitor S31‐201 (Figure 6H,I). These results confirmed that IL‐22 promotes DC maturation and DC‐induced Th17 cell proliferation through the IL‐6/STAT3 pathway.

## 4 DISCUSSION

In our study, ApoE^−/−^ and age‐matched C57BL/6J mice were killed and then analysed at weeks 0, 4, 8 and 12. We observed that IL‐22R1 is expressed in aortic tissue and that Th22 cells are the major producers of IL‐22 during the progression of AS. In addition, Th22 cell numbers and IL‐22 levels substantially increased in mice with AS. These results indicated that Th22 cells have a role in the development of AS. Then, we used a randomized, controlled study of animal models to investigate the role of IL‐22 in AS development. We observed a larger plaque size, increased infiltration of T cells and macrophages, a higher collagen content and lower expression of SMC α‐actin in mice treated with rIL‐22 compared to those treated with PBS. We also observed the opposite results in mice treated with IL‐22 mAb. These findings suggest that by secreting IL‐22, Th22 cells play a role in plaque formation by stimulating the infiltration of T cells and macrophages and the dedifferentiation of contractile SMCs into synthetic SMCs, changes that contribute to the aggravation of AS. Importantly, we showed that mice treated with IL‐22 displayed elevated Th17 cell and DC numbers, whereas the Th1 cell numbers were not altered. In addition, we detected increases in IL‐6 levels and STAT3 phosphorylation in rIL‐22–treated ApoE^−/−^ mice. When circulating IL‐22 was blocked, the results were reversed. Taken together, these data demonstrate that the proatherogenic role of IL‐22 in the development of AS is associated with IL‐6/STAT3 activation and DC induction of Th17 cell proliferation.

Other studies have also evaluated the role of Th22 cells/IL‐22 in AS. Immunostaining results have demonstrated the existence of IL‐22 in plaques from both asymptomatic and symptomatic patients, and IL‐22 immunostaining was shown to be 7.15‐fold higher in symptomatic patients than in asymptomatic patients.^36^ A clinical study involving patients with AS detected Th22 cells in the peripheral blood by flow cytometry. In addition, percentage of Th22 cells (CD4^+^IFN‐γ^−^IL‐17^−^IL‐22^+^ cells) was notably increased in acute myocardial infarction (2.23 ± 1.58%) and unstable angina (2.09 ± 0.60%) patients compared with stable angina patients (0.93 ± 0.26%) or healthy controls (0.84 ± 0.18%).^37^ In contrast, the results of dilated cardiomyopathy (DCM) studies showed that Th22 cells may inhibit myocardial fibrosis, indicating that these cells may have a protective role in DCM.^38^ Thus, the role of Th22 cells in cardiovascular disease (CVD) needs to be further explored.

Notably, some processes previously defined as part of the Th17‐mediated immune response may also involve Th22 cells. Zenewicz observed that mice lacking IL‐22 are extremely sensitive to liver damage caused by concanavalin A (ConA), showing that the secretion of IL‐22, but not IL‐17, by Th17 cells has a protective role.^39^ Another study demonstrated that although both IL‐22 and IL‐17 can induce the production of CXC chemokines and granulocyte clone stimulation factor in the lungs, only IL‐22 can promote lung epithelial cell proliferation and strengthen the resistance to epithelial injury.^40^ When the above research was published, Th22 cells had not yet been identified, and IL‐22 was classified as a Th17 cytokine as a consequence. Thus, Th22 cells rather than Th17 cells likely mediate these protective effects. Th22 cells produce between 37% and 63% of the IL‐22 generated from all IL‐22–producing cells, while only approximately 10%‐18% of Th17 cells coexpress IL‐22 and IL‐17.^41^ Therefore, Th22 cells may have a more important role than Th17 cells in these diseases. To address this issue, we examined the dynamic frequencies of Th22 cells and IL‐22–producing Th17 cells in plaques. We observed a greater difference in Th22 cell frequencies than in IL‐22–producing Th17 cell frequencies, suggesting that Th22 cells may have a more crucial influence on AS than Th17 cells through IL‐22. Th17 cells exert their proatherogenic effects through IL‐17 rather than IL‐22. To further demonstrate the pre‐dominance of Th22 cells, we performed an intervention experiment.

Many studies have demonstrated that monocytes have a key role in AS. Monocytes can infiltrate plaques, become activated and develop into macrophages that can accumulate in the vascular wall. Furthermore, these cells engulf high levels of lipids and eventually form foam cells that accelerate atherosclerotic plaque formation.^42^ T cells have been the focus of intense research over the last 20 years because of their potential role in the development and progression of AS, as stated above. Therefore, T cells (CD3^+^) and macrophages (CD68^+^) were tested in an intervention experiment. We observed that rIL‐22 treatment substantially increased plaque sizes in ApoE^−/−^ mice, and this increase was accompanied by increased T cell and macrophage infiltration, whereas the IL‐22 mAb treatment strongly reduced plaque size and T cell and macrophage infiltration. These results confirmed that IL‐22 can exacerbate AS.

The migration and proliferation of SMCs are crucial in the development of AS. Reports have shown that the loss of contractile proteins, especially SMC α‐actin, is central to the migration and proliferation of SMCs in the intima.^43^, ^44^, ^45^ In addition, medial SMCs that migrate into the intima decrease their contractile properties to become synthetic SMCs, which produce extracellular matrix proteins during plaque formation.^46^ In our study, mice treated with rIL‐22 exhibited increased collagen contents as well as reduced SMC α‐actin expression. Mice treated with the anti–IL‐22 neutralizing mAb exhibited the opposite results. These findings suggest that IL‐22 affects the dedifferentiation of SMCs, a conclusion that is consistent with the research of Sara Rattik, who observed decreased expression of genes associated with contraction after stimulating SMCs with IL‐22 in vitro.^34^

Based on our study showing that IL‐22 could exacerbate AS development and elevate T‐cell numbers in mice, we further assessed proatherogenic inflammatory factors and the primary downstream pathway of IL‐22 to investigate possible mechanisms by which IL‐22 exacerbates AS. We discovered notable increases in the IL‐6 concentration and the levels of Th17 cells in rIL‐22–treated ApoE^−/−^ mice. IL‐6 is an upstream inflammatory cytokine that contributes to AS by promoting the proliferation and differentiation of lymphocytes and by activating downstream inflammatory cytokines and the hypothalamic‐pituitary‐adrenal axis.^47^ IL‐6 secreted by APCs, especially mature DCs, is crucial in promoting the differentiation of Th17 cells.^48^ Therefore, we next quantified the mature DCs and examined whether the enhancement of IL‐6 expression and mature DCs via pre‐treatment with IL‐22 may increase Th17 cell proliferation. The JAK/STAT pathway plays important roles in the specialization, proliferation and apoptosis of various types of cells, including T cells and DCs. Previous studies have demonstrated that IL‐22 enhances the proliferation and migration of pulmonary SMCs in the airway via STAT3‐, ERK1/2‐, MAPK‐ and NF‐kB‐dependent pathways.^49^, ^50^ Unsurprisingly, we observed increased STAT3 protein phosphorylation in rIL‐22–treated mice. Therefore, we concluded that IL‐22 promotes DC maturation and DC‐induced Th17 cell proliferation through the IL‐6/STAT3 pathway. To further support this conclusion, we validated our results in vitro. Consistent with our findings in animal experiments, BMDCs stimulated with rIL‐22 and ox‐LDL differentiated into mature DCs more efficiently than untreated cells. We used the supernatant collected from mature DCs to simulate a coculture of DCs and CD4^+^ T cells and confirmed that IL‐22 could promote DC‐induced Th17 proliferation under high‐fat conditions. Moreover, Th17 proliferation and STAT3 phosphorylation were neutralized by an IL‐6 mAb and a STAT3 inhibitor. This finding suggests that the IL‐6/STAT3 pathway mediates DC‐induced Th17 proliferation. However, we cannot exclude the possibility that other cell signalling pathways acting on IL‐22 aggravated the progression of AS.

Taken together, our results demonstrated that among the known CD4^+^ cells, Th22 cells are the major source of IL‐22, which plays a major role in AS. IL‐22 activates IL‐6/STAT3, increases DC‐induced Th17 cell proliferation and stimulates SMC dedifferentiation into a synthetic phenotype, ultimately promoting the development of AS. Our findings provide new insights into the mechanism by which Th22 cells promote AS and suggest a potential target for the treatment of AS.

## CONFLICT OF INTEREST

The authors declare that they have no conflicts of interest.

## AUTHOR CONTRIBUTIONS

LS and QJ designed and performed the experiments, analysed the data and wrote the manuscript. LL and YS helped to guide and revise the manuscript. ZL and YJ helped to construct the animal models and carry out the interventions. TZ and YX performed the flow cytometric analysis. ZY and YL analysed the RT‐PCR and ELISA data and created some of the graphs. JL and XH helped to analyse the immunohistochemistry and Western blotting data. QQ and TL helped to collect tissue and blood samples. YL planned and supervised this study. All authors have approved the final version of the manuscript.

## Data Availability

I confirm that my article contains a Data Availability Statement, even if no data are available (list of sample statements), unless my article type does not require one (*eg* Editorials, Corrections and Book Reviews). I confirm that I have included a citation for available data in my references section unless my article type is exempt.
